# Family Complexity and Later-Life Wealth: A Comparative Perspective Across European Countries

**DOI:** 10.1007/s10680-026-09781-x

**Published:** 2026-08-20

**Authors:** Davide Gritti, Stefani Scherer

**Affiliations:** Department of Sociology and Social Research, University of Trento, Via Verdi, 26 - 38122 Trento, Italy

**Keywords:** Family complexity, Wealth accumulation, Sequence analysis, Family trajectories, Life course

## Abstract

This paper investigates how the complexity of individual family trajectories relates to personal net wealth in later life, in comparative perspective. Using retrospective and prospective SHARE data (waves 1–7, 2004–2017), we analyse cohorts born between the 1930s and 1960s across ten European countries, grouped into Scandinavian, Continental, and Southern clusters. We derive a measure of family complexity from sequence analysis and estimate generalised additive models to assess its non-linear association with personal net wealth at age 50 and beyond, separately for women and men, and examine whether this association persists once qualitative trajectory patterns (traditional, modern, postponed, dissolution) are accounted for. Results show that the complexity–wealth association is neither linear nor universal. In Scandinavian and Continental countries, predicted wealth peaks at moderate complexity and is lower at both extremes: in Scandinavia, the high-complexity penalty is concentrated among men, while women face sharper disadvantage at low complexity; in Continental countries, women are penalised at both ends, whereas men face a significant penalty only at low complexity, with no significant association at the upper end once education is controlled. In Southern countries, complexity is monotonically and positively associated with wealth for both sexes, with the steepest gradient among women. The complexity–wealth gradient is largely unaffected by conditioning on qualitative trajectory patterns, indicating that its consequences are not reducible to specific biographical configurations. Educational sorting attenuates but does not eliminate these associations. Our findings underscore the importance of institutional context and social stratification in shaping the long-term economic consequences of family diversification.

**Supplementary Information:**

The online version contains supplementary material available at https://doi.org/10.1007/s10680-026-09781-x.

## Introduction

A large body of research has examined the wealth consequences of specific demographic events, most notably marriage and divorce (Killewald et al., 2023; Lersch, 2017; Vespa & Painter, 2011; Wilmoth & Koso, 2002; Zagorsky, 2005). By design, however, this event-centred literature has limited leverage on how entire family trajectories relate to wealth; exceptions include recent trajectory-based approaches that examine heterogeneity in family life courses and its wealth implications (Kapelle & Vidal, 2022; Kapelle & Rowold, 2025). Moreover, evidence on the wealth implications of demographic events remains largely confined to single-country studies, particularly from the United States, Germany, and the United Kingdom (as an exception, see for instance Kapelle et al., 2025).

These features have contributed to a persistent disconnect with the large literature on family diversification, which has documented how family life courses have transformed across cohorts and institutional contexts (Aisenbrey & Fasang, 2010; Billari & Liefbroer, 2010; Elzinga & Liefbroer, 2007; Elzinga, 2010; Halpern-Manners et al., 2015; Mayer, 1989; Thomson, 2014). A central insight from this body of work is the rise of family complexity, understood as within-trajectory differentiation in terms of the number of transitions and the diversity of states experienced (Rindfuss et al., 1987; Van Winkle, 2019; Van Winkle & Fasang, 2021; Westerman et al., 2023). Yet, while research on the determinants of family complexity has expanded, far less attention has been paid to its consequences, despite early calls for such an investigation (Elzinga & Liefbroer, 2007).

This study bridges these strands of research by adopting a cross-national, life-course perspective on how diversification in family biographies relates to personal wealth in midlife and old age, and whether this association varies across institutional contexts. We ask: *how are complex family trajectories associated with wealth*,* and to what extent is this association universal or context-dependent across Europe?*

We address this question using data from the Survey of Health, Ageing and Retirement in Europe (SHARE), combining prospective information on wealth with retrospective biographies. We reconstruct annual family trajectories between ages 15 and 50 and relate them to personal net wealth observed at age 50 and above among cohorts born between the 1930 s and the 1960 s in ten European countries. The main explanatory variable is a continuous measure of family complexity derived from sequence analysis, capturing within-trajectory differentiation in terms of transitions and state diversity. For coherence with comparative research on family change and its institutional patterning, we group countries into three clusters: Scandinavian (Sweden, Denmark), Continental (Austria, Germany, France, Switzerland, Belgium), and Southern (Spain, Italy, Greece).

## Background

Understanding how diversification in family trajectories might shape later-life wealth is challenging because diversification refers to analytically distinct forms of heterogeneity in family life courses. We first clarify the distinction between diversification between individuals (destandardisation) and diversification within individual biographies (differentiation), and locate family complexity within this conceptual map. We then outline the economic mechanisms through which specific family trajectories can facilitate or hinder wealth accumulation, motivating why complexity may be consequential for later-life wealth and why the association may be non-linear. Third, we discuss how social stratification shapes both exposure to complex trajectories and wealth outcomes, specifically positioning education as a key confounder in the relationship between family complexity and wealth. Finally, we discuss why the meaning and potential implications of family complexity may differ across European institutional contexts, and why these differences may be gendered.

### The Diversification of Family Trajectories

The concept of diversification is closely linked to broader accounts of how human lives are shaped by structural and cultural change (Mayer, 1989; Esping-Andersen & Billari, 2015; Goldscheider et al., 2015). Whereas cohorts entering adulthood in the post-war decades typically followed early, stable, and relatively homogeneous family formations (Billari & Liefbroer, 2010; Sobotka & Berghammer, 2021), more recent cohorts have experienced increasingly diverse and turbulent family and work lives (Kohli, 2007; Ramos, 2019). Within the life-course tradition, an influential distinction captures these shifts: diversification between life courses versus diversification within life courses (Brückner & Mayer, 2005; Elzinga & Liefbroer, 2007), often labelled destandardisation and differentiation, respectively (Zimmermann et al., 2024).

Destandardisation refers to declining uniformity across individuals and cohorts, reflected in a broader range of partnership and fertility pathways. Related notions such as de-institutionalisation emphasise the weakening of traditional norms and the diffusion of new arrangements, exemplified by cohabitation becoming an accepted pathway to family formation (Cherlin, 2004; Hofäcker & Chaloupková, 2014; Perelli-Harris & Lyons-Amos, 2015). Differentiation, by contrast, captures the internal turbulence of biographies: the number, ordering, and reversibility of transitions between family states over the life course (Brückner & Mayer, 2005). This within-trajectory dimension is closely captured by family complexity. In sequence-analytic work, complexity has been formalised as a combination of the frequency of transitions and the diversity of states experienced, thereby summarising volatility and unpredictability in family biographies (Elzinga, 2010; Gabadinho et al., 2011).

### Consequences of Family Diversification for Wealth

Diversified family trajectories are likely consequential for wealth because events and dynamics in the family domain structure opportunities for saving, losing, investing, and risk-sharing over the life course. In line with the life-course paradigm and cumulative advantage perspectives, later-life outcomes can be understood as the result of prior events and trajectories that shape resources and constraints over time (Halpern-Manners et al., 2015). The relationship between demographic behaviours—such as union formation and dissolution—and wealth is typically explained through the economic mechanisms embedded in these events (Killewald et al., 2017; Lersch et al., 2022), which can either enhance or deplete wealth.

Early and stable family formation has been linked to stronger opportunities for saving and investment, partly because stable partnerships support long-term planning and allow couples to benefit from economies of scale and joint investments (Bennett, 2013; Kapelle & Lersch, 2020; Lersch et al., 2022). Such trajectories also avoid the direct and indirect financial costs associated with union dissolution (Avellar & Smock, 2005; Killewald et al., 2023; Uunk, 2004). By contrast, trajectories marked by delayed or less stable unions may undermine wealth accumulation through several channels. Cohabitation, relative to marriage, is often associated with lower economic integration between partners (Vitali & Fraboni, 2022; Hiekel et al., 2014; Lauer & Yodanis, 2011), while dissolution entails legal and relocation costs and may disrupt savings and investment trajectories. Longer spells outside stable partnerships can also limit opportunities for joint investments and the accumulation of durable assets. Fertility adds a further layer. Raising children increases household consumption and can constrain saving, yet parenthood may also incentivise precautionary saving and long-term investments as parents seek to secure their family’s future (Lersch, 2017; Vespa & Painter, 2011). These mechanisms underscore why single demographic events matter for wealth, but they also motivate a trajectory-based approach that captures exposure to repeated changes and heterogeneous family states across adulthood.

In this study, family complexity captures such exposure to within-trajectory differentiation in an integrative way: a higher number of transitions and greater state diversity can reflect repeated reconfigurations of partnership arrangements, periods of singlehood, and shifting family responsibilities, all of which may hinder long-term financial planning (Fulda & Lersch, 2018). Unpredictability may be particularly consequential for investments that require sustained commitments, such as homeownership. At the same time, the association need not be linear: very low complexity (e.g., prolonged singlehood) may also limit opportunities for joint accumulation, whereas very high complexity may concentrate multiple wealth-depleting mechanisms.

Further, a growing body of evidence indicates that family life courses have gender-differentiated consequences for wealth (Kapelle & Rowold, 2025; Lersch et al., 2022). Events such as marriage and, even more so, divorce tend to penalise women more than men. While both genders benefit from marriage-related wealth gains, women’s gains are typically smaller (Lersch, 2017). Following separation, both men and women may experience sharp declines in personal wealth (Kapelle & Baxter, 2021), but women often start from lower wealth levels and recover less over the longer term (Kapelle, 2022). These gendered consequences contribute to the gender wealth gap (Ruel & Hauser, 2013; Schneebaum et al., 2018), which is rooted in cumulative disadvantages in employment trajectories (Nutz & Gritti, 2022), occupational positions (Waitkus & Minkus, 2021), and intergenerational wealth transfers (Trinh, 2024).

Finally, while focusing on the complexity of entire family trajectories can reduce concerns about short-term reverse causality tied to single events, it does not allow for causal claims. Unobserved changes in economic resources across the life course may shape family behaviours and thereby influence family trajectories (Lersch, 2025). Wealth and family complexity may thus co-evolve over time, and we interpret our findings as descriptive associations rather than causal effects.

### Education as a Form of Selection into Family Diversification and Wealth

Family diversification is not a universal postmodern condition but a socially stratified and context-dependent process. Zimmermann and Konietzka (2018) show that destandardisation is often more pronounced among the lower educated, consistent with accounts linking family instability to economic insecurity and unfavourable marriage markets at the bottom of the educational hierarchy, even though higher-educated groups may pioneer new family patterns. At the level of specific trajectories, higher-educated women and men frequently lead ‘modernised’ and postponement patterns in several European settings.

These patterns matter for the present study because education is also strongly linked to wealth through multiple pathways, including occupational opportunities, income trajectories, financial literacy, and social origins (Hansen & Toft, 2021; Killewald et al., 2017). Education therefore emerges as a key confounder in the relationship between family complexity and wealth. Apparent wealth advantages or disadvantages associated with complex trajectories may partly reflect selection into both family trajectories and wealth accumulation processes. Accounting for education is thus essential to assess whether the observed complexity–wealth association is robust to educational sorting.

### Institutional Pathways to Family Diversification

Comparative research documents substantial cross-national variation in both destandardisation and within-trajectory differentiation, and shows that these differences are systematically patterned by broad institutional contexts (Elzinga & Liefbroer, 2007; Van Winkle, 2017, 2019; Van Winkle & Fasang, 2021; Zimmermann et al., 2024). Regime-informed accounts commonly distinguish a Nordic/social-democratic context—where diversification and within-trajectory differentiation tend to be comparatively pronounced—from a Mediterranean context, where traditional family norms and more limited public support for alternative family forms are associated with later and more selective departures from standard pathways; Continental countries typically occupy an intermediate position, with more gradual shifts and moderate increases in life-course diversity (Van Winkle, 2019; Elzinga & Liefbroer, 2007; Zimmermann et al., 2024). Building on this literature, we organise empirical analysis around three country clusters that map onto these well-established contrasts.

Against this backdrop, the same aggregate level of complexity may reflect partially distinct paths to diversification across contexts. In some settings, diversification is more closely tied to the early diffusion and normalisation of alternative partnership forms. In others, it is more strongly linked to delayed and selective departures from traditional pathways, with longer spells outside stable unions and a more concentrated incidence of disruption. As a result, similar levels of complexity can correspond to different underlying configurations of states and transitions, and therefore to different bundles of economic mechanisms operating. More generally, comparative work suggests that cross-national differences concern not only how common diversified trajectories are, but also how diversification unfolds—i.e., which transitions become socially legitimate, and how strongly family change departs from age-graded sequencing norms (Elzinga & Liefbroer, 2007; Van Winkle, 2019; Zimmermann et al., 2024). This implies that the wealth implications of a given level of family complexity need not be uniform across Europe, because institutional environments shape the scope for buffering the consequences of family complexity.

One institutional dimension that helps interpret these cross-context differences is the orientation of family policies towards familisation versus individualisation/defamilisation. Comparative evidence suggests that more individualising policy packages—by supporting independent living arrangements and buffering family-related risks outside the couple—are associated with higher levels of within-trajectory differentiation, whereas more familising arrangements tend to be linked to lower levels of complexity (Van Winkle, 2019). This perspective is relevant not only for understanding why complexity is more prevalent in Nordic contexts and less common in Mediterranean ones, but also for interpreting its potential economic implications. Where individualisation is stronger, complex trajectories may be more ‘institutionally accommodated’ and their economic risks partly buffered. This might be the case especially for women, for whom policies supporting employment continuity and individual resources can mitigate the costs of partnership instability. By contrast, in more familising contexts—where complexity is less prevalent and family dependence is higher—the same degree of complexity may translate into sharper economic consequences because fewer institutional supports offset the consequences of disrupted or non-standard family careers.

## Data and Methods

### Data

We draw on data from the Survey of Health, Ageing and Retirement in Europe (SHARE, release 7.1.0) (Börsch-Supan et al., 2013), a cross-national panel survey conducted biennially since 2004/2005 among individuals aged 50 and over and their partners. To examine wealth and reconstruct family histories, we use data from waves 1, 2, 4, 5, 6, and 7 for current wealth and sociodemographic characteristics, and from the retrospective modules SHARELIFE (waves 3 and 7) for full partnership and fertility histories. Given the targeted age group of SHARE respondents (50+), and the combination of prospective information on wealth in the standard module with retrospective information on family trajectories from the SHARELIFE modules, we are able to link wealth in later life to family trajectories with their wealth-enhancing and wealth-depleting mechanisms, occurring between the ages of 15 and 50.

We focus on ten countries for which both wealth and retrospective family-history data are available: Austria, Germany, Sweden, Spain, Italy, France, Denmark, Greece, Switzerland, and Belgium. Following comparative life-course research documenting regime-patterned differences in family diversification and within-trajectory differentiation, we organise countries into three clusters: Scandinavian (Sweden, Denmark), Continental (Austria, Germany, France, Switzerland, Belgium), and Southern (Spain, Italy, Greece) (Elzinga & Liefbroer, 2007; Zimmermann & Konietzka, 2018; Van Winkle, 2019).

### Sample

We restrict our analysis to individuals born between 1932 and 1962 in ten countries, including those who are either currently single or in a couple. Individuals living with other family members — such as adult children or other relatives — are excluded, as it is not possible to assess their personal net wealth. These restrictions allow us to capture cohorts with varying exposure to family complexity across contexts, while avoiding the inclusion of very old individuals (maximum age is 85) who may have already decumulated a substantial portion of their wealth. To minimise the temporal distance between the life-course window used to construct family trajectories (ages 15–50) and the measurement of wealth, we retain only the first available observation for each respondent. The final analytical sample is composed of 30,509 individuals. Table A1 in the Appendix summarises the sample selection process.

### Measures

#### Family Complexity

Family trajectories are operationalised as sequences of categorical states observed yearly from age 15 to age 50. The underlying information comes from the retrospective biographies collected in the SHARELIFE modules (waves 3 and 7), covering accommodation histories, partnership histories, and fertility. These biographies are harmonised and recoded into a person-year structure using the generated Job Episode Panel, which provides a convenient framework to combine retrospective spells across domains (Brugiavini et al., 2019; Orso et al., 2016). The resulting sequences include ten mutually exclusive states: living in the parental home (with or without children), single (with or without children), cohabiting (with or without children), married (with or without children), and divorced (with or without children).

To capture within-trajectory differentiation, we use the family complexity index proposed by Gabadinho et al. (2011) and adopted in recent comparative work (Van Winkle, 2017; Winkle, 2019; Westerman et al., 2023). This measure combines two components: the complexity of sequencing, measured by the number of transitions across states, and the diversity of states experienced along the trajectory, measured by Shannon’s entropy (Gabadinho et al., 2011). Higher values therefore indicate biographies characterised by more frequent changes and a broader repertoire of family states. The index ranges from 0 (no transitions and no entropy) to 1 (maximum possible transitions and entropy). For descriptive purposes, we additionally classify respondents into three complexity groups based on their standardised distance from the overall mean: low complexity (≤ − 0.5 SD), medium complexity (between − 0.5 and + 0.5 SD), and high complexity (≥ + 0.5 SD). These cut-off points are defined on the pooled distribution and applied uniformly across all country clusters.

#### Wealth

The outcome of interest is personal net wealth at the time of the interview, expressed in 1,000 €. In the SHARE survey, an imputed measure of household net worth is available. Household net worth is calculated as the sum of household real assets (e.g., value of the main residence, other properties, businesses, cars, net of mortgages) and net financial assets (e.g., value of bank accounts, bonds, stocks, funds, and savings, net of liabilities). For individuals in couples, we derive a measure of personal net wealth under the assumption of equal sharing between partners.

We apply a series of transformations to the original wealth variable to address the highly skewed distribution of wealth and the presence of negative values. First, all values are converted into euros adjusted to 2015 purchasing power parity (PPP) in Germany for comparability. Second, values are top- and bottom-coded at the 0.1 and 99.9 percentiles. We adopt alternative measures in robustness checks.

#### Additional Covariates

Highest level of education attained is based on recoding of the original ISCED-1997 scale into low (ISCED 0–3, up to lower secondary), middle (ISCED 3, upper secondary), and high (ISCED 4–6, up to tertiary).

Consistent with the conceptual distinction between quantitative differentiation and qualitative sequencing, we derive a small set of theory-informed ideal-typical trajectory patterns that capture qualitative differences among family biographies. Drawing on Zimmermann and Konietzka (2018) and Burgin et al. (2017), we identify four patterns that emphasise the ordering of episodes as a distinctive source of trajectory variation: (1) a traditional family formation pattern, characterised by an exit from the parental home immediately followed by marriage (with or without children); (2) a modern pattern, featuring a spell of either cohabitation or singlehood after leaving the parental home prior to marriage; (3) a postponed pattern, defined by the absence of couple formation (marriage or cohabitation) before age 35; and (4) a dissolution pattern, featuring at least one marriage ending in separation or divorce.^1^ Each respondent is binary-coded for whether their observed trajectory matches each ideal-typical trajectory pattern; the indicators are non-mutually exclusive, so a respondent may simultaneously match more than one pattern (e.g., both modern and dissolution), while those whose trajectories do not match any pattern are coded zero on all indicators.^2^ These four measures are complemented by a measure of completed fertility by age 50.

### Analytical Strategy

To investigate how family complexity relates to later-life wealth, we adopt a three-step analytical strategy aligned with the arguments outlined in the background section. All analyses are stratified by country cluster and gender.

First, we begin by describing how family complexity is distributed across institutional contexts and how the qualitative composition of ideal-typical trajectory patterns differs across levels of complexity. Using the three complexity groups defined in Sect. 3.3, we document, within each group, the prevalence of the four ideal-typical trajectory patterns and average completed fertility by age 50.

Second, we assess whether the association between family complexity and wealth varies across institutional contexts and whether it is linear or non-linear by estimating generalised additive models (GAMs). GAMs extend generalised linear models by combining conventional parametric terms with smooth, spline-based functions of selected predictors, thereby relaxing linearity assumptions and allowing for flexible, potentially non-monotonic relationships (Hastie & Tibshirani, 1987; Andersen, 2009; Schaeffer et al., 2016). In our application, the smooth term for family complexity traces how wealth varies over the full range of complexity, while age, age squared, and country indicators enter linearly. We estimate two specifications: a baseline model adjusting for age, age squared, and country fixed effects, and a second model additionally controlling for educational attainment to account for educational sorting into both family complexity and wealth. These models take the following form:

Baseline model:

1 $$\:Y=\alpha\:+f\left({x}_{complexity}\right)\:+\beta\:\left({x}_{age}\right)\:+\beta\:\left({x}_{country}\right)$$

Adjusted model:

2 $$\:Y=\alpha\:+f\left({x}_{complexity}\right)+\beta\:\left({x}_{age}\right)\:+\:\beta\:\left({x}_{country}\right)\:+\:\beta\:\left({x}_{education}\right)$$

where f(complexity) is the smooth function of standardised family complexity. To summarise whether the association is approximately linear or clearly non-linear within each cluster and gender, we report the estimated degrees of freedom (edf) of the complexity smooth: values close to 1 indicate an approximately linear relationship, while higher values indicate increasingly curved functional forms.

Third, we examine whether the observed association between family complexity and wealth persists once qualitative differences in family biographies are taken into account. We thus add the ideal-typical trajectory indicators and completed fertility as additional parametric terms to the smooth model, first one at a time and then all jointly. Rather than interpreting this step as a mediation analysis, we use it to assess whether the non-linear complexity–wealth gradient remains once qualitative sequencing patterns and fertility are held constant.

## Results

### Describing Family Complexity Across Countries

Table 2 provides empirical context for subsequent multivariate analyses by relating the quantitative stratification of family trajectories (low/medium/high complexity) to their qualitative configuration. It reports the distribution of respondents across complexity groups within each country cluster and, for each cluster–complexity cell, the prevalence of the ideal-typical trajectory patterns and completed fertility (Table 1).

**Table 1:** Sequence-based measures of family trajectories adopted in this study

Name	Construction (range)	Source
Complexity	Geometric mean of normalized sequence transitions and normalized sequence entropy (0–1)	Gabadinho et al. (2011)
Traditional trajectory	Exit from parental home immediately followed by marriage (0/1)	Zimmermann and Konietzka (2018) Burgin et al. (2017)
Modern trajectory	At least one episode of cohabitation/singlehood after leaving parental home (0/1)	Zimmermann and Konietzka (2018) Burgin et al. (2017)
Postponement trajectory	No couple formation (marriage/cohabitation) before age 35 (0/1)	Zimmermann and Konietzka (2018) Burgin et al. (2017)
Dissolution trajectory	At least one marriage dissolution (divorce or separation) between age 18 and 50 (0/1)	Zimmermann and Konietzka (2018) Burgin et al. (2017)
Completed fertility	Children had by age 50 (0+)	

**Table 2:** Family complexity groups and prevalence of ideal-typical trajectory patterns and completed fertility, by country cluster

Country cluster	Complexity level	*N*	Share within cluster (%)	Traditional (%)	Modern (%)	Postponement (%)	Dissolution (%)	Completed fertility
Scandinavian	Low	987	16.21	36.27	10.84	14.49	0.91	2.12
	Medium	2,428	39.88	39.00	46.29	5.77	4.28	2.21
	High	2,674	43.92	0.00	82.09	2.88	44.05	2.32
Continental	Low	3,891	26.56	48.65	3.37	16.22	0.44	1.86
	Medium	6,514	44.46	66.23	20.16	5.28	6.08	2.10
	High	4,247	28.99	0.00	61.97	4.36	57.15	2.02
Southern	Low	3,251	33.28	55.83	0.49	19.56	0.22	1.83
	Medium	5,683	58.18	89.41	5.67	4.82	1.74	2.13
	High	834	8.54	0.00	57.91	8.99	44.36	1.90
Overall		30,509		47.23	27.28	8.21	15.10	2.06

“Share within cluster (%)” reports the distribution of respondents across low/medium/high complexity and sums to 100 within each country cluster. Pattern columns report the percentage of respondents within each cluster × complexity cell who display the corresponding trajectory feature. Because patterns are coded as non-mutually-exclusive binary indicators, row percentages need not sum to 100. “Completed fertility” is the mean number of children by age 50. The “Overall” row reports pooled values for the full sample. Countries in clusters: Scandinavia (Sweden, Denmark); Continental (Austria, Germany, France, Switzerland, Belgium); Southern (Spain, Italy, Greece)

Data: SHARE W1–W7. *N* = 30,509

Consistent with evidence discussed in Sect. 2.1, family complexity is unevenly distributed across contexts. High complexity is comparatively common in the Scandinavian cluster (44%) and remains sizeable in the Continental cluster (29%), whereas it is much less frequent in the Southern cluster (9%), where most individuals fall into the medium-complexity group (58%). Low complexity is most prevalent in Southern countries (33%) and least prevalent in Scandinavian ones (16%).

#### Low Complexity

Low complexity is primarily associated with traditional trajectories in all clusters, with the highest prevalence in Southern countries (56%), followed by Continental (49%) and Scandinavian countries (36%). Modern trajectories are rare at low complexity—especially in Continental (3%) and Southern countries (0%)—and dissolution is virtually absent across contexts (≤ 1%). Postponement is most visible in the low-complexity group in Southern countries (20%), compared with Scandinavian (14%) and Continental countries (16%).

#### Medium Complexity

The medium-complexity group differs markedly across clusters. In Scandinavian countries, it combines substantial prevalence of both traditional (39%) and modern trajectories (46%), with relatively limited dissolution (4%). In Continental countries, medium complexity remains strongly anchored in traditional trajectories (66%), while modern trajectories are less prominent (20%) and dissolution is modest (6%). In Southern countries, medium complexity is overwhelmingly traditional (89%), with only limited prevalence of modern trajectories (6%) and dissolution (2%). Postponement is relatively uncommon across all three clusters at medium complexity (about 5–6%).

#### High Complexity

At high complexity, traditional trajectories virtually disappear in all clusters (0%), while modern and dissolution patterns become dominant features. Modern trajectories are particularly prevalent in the Scandinavian cluster (82%), followed by the Continental (62%) and Southern clusters (58%). Dissolution trajectories also rise sharply with high complexity, reaching 57% in the Continental cluster and 44% in the Scandinavian cluster; in the Southern cluster, dissolution is similarly widespread among the highly complex (44%) despite being rare at lower complexity.

Completed fertility varies only modestly across complexity levels. In Scandinavian countries, it increases slightly from low (2.1 children) to high complexity (2.3). In Continental and Southern countries, it peaks at medium complexity (about 2.1) and is lower among the highly complex (2.0 and 1.9, respectively).

Overall, the prevalence of family complexity differs sharply across institutional contexts: high complexity is much more common in Scandinavian and Continental countries than in Southern ones. Within each context, moreover, the same level of complexity maps onto different qualitative configurations of family biographies. As complexity increases, traditional trajectories recede and modern and dissolution patterns become more salient, but the composition of the medium- and high-complexity groups—i.e., which ideal-typical trajectories tend to generate complexity—varies across clusters.

### The Association Between Family Complexity and Wealth

**Fig. 1:**
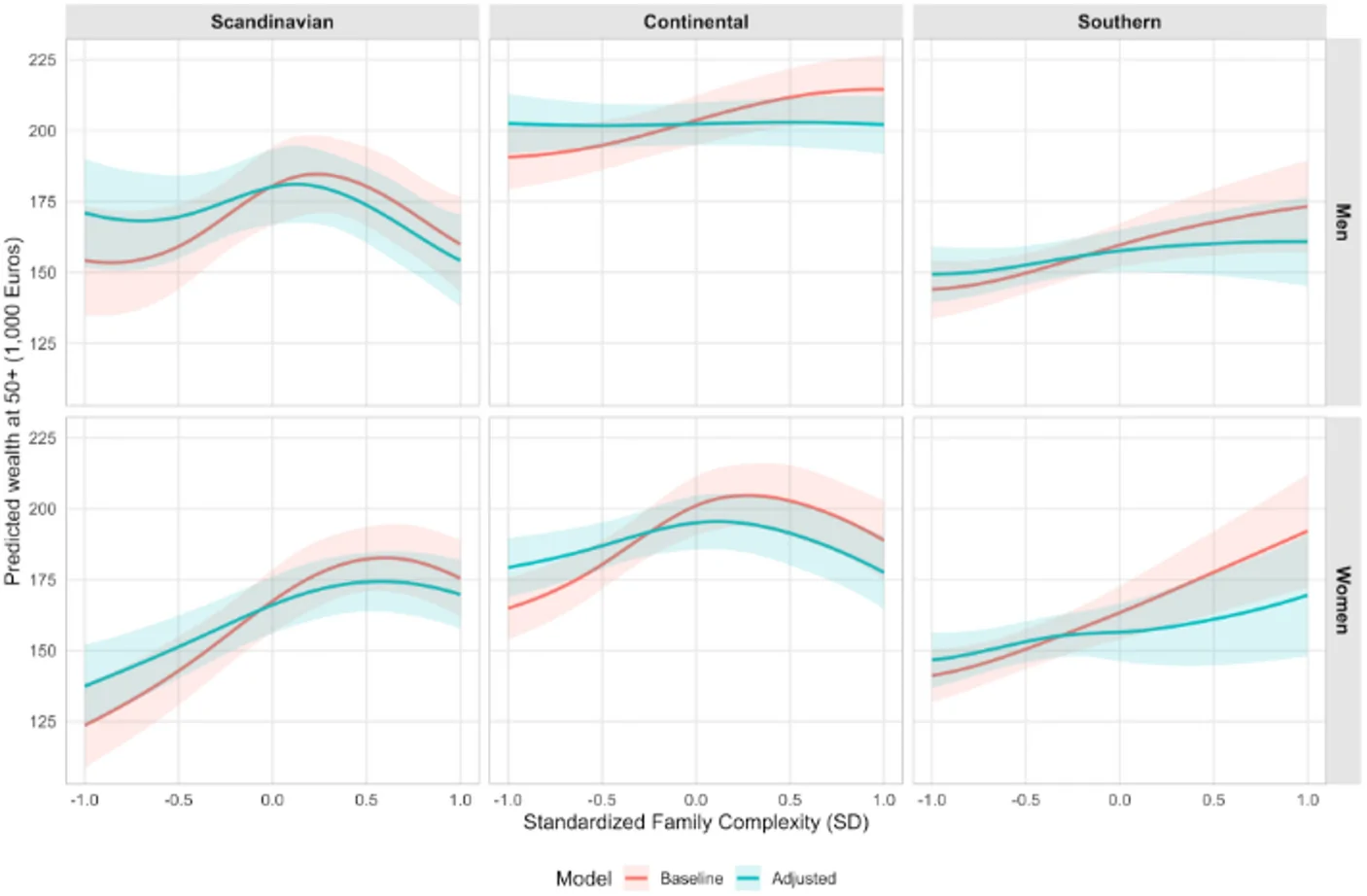
Predicted wealth at 50 + across distribution of family complexity, by country cluster and gender. Baseline models control for country and age at the interview. Adjusted models further control for educational attainment. Countries in clusters: Scandinavia (Sweden, Denmark); Continental (Austria, Germany, France, Switzerland, Belgium); Southern (Spain, Italy, Greece). Data: SHARE W1-W7. *N* = 30,509. Full tabular results in Table A3-A4-A5.

Figure 1 summarises the GAM results by plotting average predicted net wealth at ages 50 + across the observed range of standardised family complexity (− 1 to + 1 SD), separately by country cluster and gender. The baseline specification adjusts for age and country fixed effects; the education-adjusted specification additionally accounts for educational attainment. Overall, the association between family complexity and wealth is present, but neither consistently linear nor uniform across institutional contexts.

In the Scandinavian cluster, predicted wealth follows a clearly curvilinear profile, especially among men. In the baseline model, it rises from about €155k at low complexity (− 1 SD) to a peak of roughly €185k around slightly above-average complexity (≈ + 0.2 SD), and then declines at higher complexity. After adjusting for education, the downturn becomes more visible, with predicted wealth falling to around €150k at + 1 SD. Among Scandinavian women, predicted wealth increases more gradually with complexity and then levels off: in the baseline model it rises from roughly €125k at − 1 SD to about €175k at + 1 SD (peaking around €185k near + 0.6 SD), while the adjusted profile remains upward sloping but flatter (approximately €135k to €170k).

In the Continental cluster, the baseline model suggests only a modest positive gradient for men—from about €190k at − 1 SD to about €215k at + 1 SD—but once education is included, the adjusted profile is flat at the upper end of the distribution, while a significant low-complexity penalty persists (− 21k, *p* < 0.001). For women in Continental countries, the relationship is distinctly non-linear in both specifications. Predicted wealth peaks around average complexity (baseline: about €200k near + 0.3 SD; adjusted: about €195k near + 0.1 SD) and is lower at both tails; at high complexity (+ 1 SD), predicted wealth declines to around €190k in the baseline model and to roughly €180k in the education-adjusted model.

The Southern cluster stands out for its comparatively monotonic profile. Predicted wealth increases with complexity for both men and women, and the gradient remains positive—though attenuated—after adjustment for education. Among men, predicted wealth rises from about €145k at − 1 SD to around €175k at + 1 SD in the baseline model, compared to roughly €150k to €160k once education is controlled. Among women, the gradient is steeper: from approximately €140k to €190k in the baseline model, and from about €145k to €170k in the adjusted model.

Full tabular results, including the smooth-term diagnostics for family complexity, are reported in Tables A3–A5 in Appendix. The estimated degrees of freedom (edf) of the complexity smooth confirm that the association is markedly non-linear in most specifications, with edf values above 1. The smooth term is statistically significant for women in all clusters and specifications, with the exception of the education-adjusted model for Southern women (*p* = 0.093), whereas for men it is significant in the Scandinavian cluster but not in the Continental and Southern clusters once education is controlled, consistent with Fig. 1.

### Ideal-Typical Trajectory Patterns as Correlates of the Family Complexity–Wealth Association

Section 4.1 showed that similar levels of family complexity correspond to different configurations of ideal-typical trajectory patterns across institutional contexts. We now examine whether the association between family complexity and wealth persists once these qualitative differences in sequencing are taken into account. Tables 3, 4 and 5 report contrasts derived from GAM models that additionally condition on the ideal-typical trajectory indicators and completed fertility, first one at a time and then all jointly. Linear associations between the ideal-typical trajectory indicators and completed fertility are also reported.

**Table 3:** Differences in average predicted wealth (1,000 €) relative to medium family complexity, adjusted for ideal-typical trajectory indicators. Scandinavian cluster

Gender		Adjusted for trajectories				Trajectory association	
		Δ Low–Med	Sig.	Δ High–Med	Sig.	β trajectory (SE)	Sig.
Men	Traditional	−15.1 [−34.6, 4.4]		−15.4 [−29.7, −1.0]	*	1.17 (11.42)	
	Modern	−12.3 [−31.4, 6.9]		−14.4 [−28.6, −0.2]	*	21.14 (10.27)	*
	Postponement	−14.9 [−34.2, 4.5]		−15.3 [−29.7, −1.0]	*	12.35 (15.53)	
	Dissolution	−14.6 [−33.9, 4.7]		−15.0 [−29.3, −0.7]	*	−12.73 (13.29)	
	Completed fertility	−14.8 [−34.0, 4.5]		−15.1 [−29.4, −0.8]	*	−5.64 (3.66)	
	All trajectories	−7.3 [−18.0, 3.4]		−12.0 [−23.0, −0.9]	*		
Women	Traditional	−39.8 [−54.4, −25.2]	***	−6.2 [−18.8, 6.4]		17.92 (10.48)	.
	Modern	−33.1 [−45.3, −20.9]	***	−5.0 [−15.7, 5.7]		28.92 (8.55)	***
	Postponement	−37.0 [−51.1, −23.0]	***	−5.4 [−17.6, 6.8]		−45.89 (17.67)	**
	Dissolution	−36.4 [−49.0, −23.7]	***	−6.1 [−17.1, 4.9]		−44.55 (10.79)	***
	Completed fertility	−36.4 [−50.0, −22.7]	***	−5.3 [−17.1, 6.5]		3.88 (3.33)	
	All trajectories	−37.9 [−51.9, −24.0]	***	−6.4 [−18.5, 5.6]			

Δ Low–Med and Δ High–Med report differences in average predicted wealth (1,000 €) relative to the medium-complexity group, derived from GAMs using the delta method over the observed covariate distribution within each cluster × gender group. Models include age, country fixed effects, educational attainment, and the respective ideal-typical trajectory indicator (or all indicators jointly in the last row). Baseline Δ values (without trajectory controls) are reported in Table 6. β trajectory reports the parametric coefficient of the trajectory indicator in the GAM. Complexity levels: low (≤ − 0.5 SD), medium (− 0.5 to + 0.5 SD), high (≥ + 0.5 SD). 95% confidence intervals in brackets. Sig.:. *p* < 0.10, * *p* < 0.05, ** *p* < 0.01, *** *p* < 0.001

Data: SHARE W1-W7. *N* = 6,089

**Table 4:** Differences in average predicted wealth (1,000 €) relative to medium family complexity, adjusted for ideal-typical trajectory indicators. Continental cluster

Gender		Δ complexity, adj. for trajectories				Trajectory association	
		Δ Low–Med	Sig.	Δ High–Med	Sig.	β trajectory (SE)	Sig.
Men	Traditional	−23.7 [−35.8, −11.5]	***	10.4 [−1.8, 22.6]	.	13.37 (8.03)	.
	Modern	−21.6 [−32.3, −10.8]	***	10.5 [−0.5, 21.6]	.	−2.68 (8.54)	
	Postponement	−19.7 [−28.7, −10.7]	***	10.7 [0.7, 20.7]	*	23.26 (11.34)	*
	Dissolution	−22.2 [−33.1, −11.3]	***	10.3 [−0.9, 21.5]	.	−13.31 (11.20)	
	Completed fertility	−19.9 [−29.0, −10.9]	***	10.3 [0.3, 20.3]	*	−5.63 (2.79)	*
	All trajectories	−24.4 [−36.4, −12.4]	***	9.4 [−2.6, 21.4]			
Women	Traditional	−38.7 [−49.4, −28.0]	***	−18.4 [−30.5, −6.3]	**	17.38 (8.05)	*
	Modern	−37.4 [−47.8, −26.9]	***	−17.3 [−29.2, −5.4]	**	−0.14 (8.31)	
	Postponement	−37.2 [−47.6, −26.9]	***	−17.3 [−29.1, −5.5]	**	13.94 (12.57)	
	Dissolution	−38.0 [−48.3, −27.7]	***	−18.1 [−29.8, −6.3]	**	−35.93 (10.10)	***
	Completed fertility	−37.3 [−47.7, −26.9]	***	−17.3 [−29.2, −5.4]	**	−0.48 (2.61)	
	All trajectories	−39.6 [−50.2, −29.0]	***	−19.1 [−31.2, −7.1]	**		

Δ Low–Med and Δ High–Med report differences in average predicted wealth (1,000 €) relative to the medium-complexity group, derived from GAMs using the delta method over the observed covariate distribution within each cluster × gender group. Models include age, country fixed effects, educational attainment, and the respective ideal-typical trajectory indicator (or all indicators jointly in the last row). Baseline Δ values (without trajectory controls) are reported in Table 6. β trajectory reports the parametric coefficient of the trajectory indicator in the GAM. Complexity levels: low (≤ − 0.5 SD), medium (− 0.5 to + 0.5 SD), high (≥ + 0.5 SD). 95% confidence intervals in brackets. Sig.:. *p* < 0.10, * *p* < 0.05, ** *p* < 0.01, *** *p* < 0.001

Data: SHARE W1-W7. *N* = 14,652

**Table 5:** Differences in average predicted wealth (1,000 €) relative to medium family complexity, adjusted for ideal-typical trajectory indicators. Southern cluster

Gender		Δ complexity, adj. for trajectories				Trajectory association	
		Δ Low–Med	Sig.	Δ High–Med	Sig.	β trajectory (SE)	Sig.
Men	Traditional	−7.0 [−18.1, 4.0]		16.4 [0.7, 32.1]	*	3.00 (7.62)	
	Modern	−7.6 [−18.5, 3.3]		15.4 [−0.1, 31.0]	.	−11.88 (11.14)	
	Postponement	−6.6 [−17.3, 4.1]		17.3 [2.1, 32.4]	*	−1.88 (10.06)	
	Dissolution	−6.3 [−17.0, 4.5]		17.7 [2.6, 32.9]	*	15.93 (17.51)	
	Completed fertility	−3.3 [−11.3, 4.6]		17.3 [6.9, 27.7]	**	−3.57 (2.76)	
	All trajectories	−7.0 [−18.2, 4.2]		16.0 [0.1, 32.0]	*		
Women	Traditional	−16.7 [−25.7, −7.8]	***	40.5 [20.8, 60.3]	***	2.93 (8.35)	
	Modern	−17.2 [−26.5, −7.8]	***	40.5 [20.3, 60.7]	***	−6.97 (15.31)	
	Postponement	−17.2 [−26.6, −7.8]	***	40.4 [19.9, 60.8]	***	5.78 (12.13)	
	Dissolution	−17.2 [−26.8, −7.7]	***	40.6 [19.7, 61.4]	***	5.08 (17.48)	
	Completed fertility	−17.9 [−27.7, −8.2]	***	39.1 [17.9, 60.2]	***	−4.91 (2.84)	.
	All trajectories	−17.9 [−27.7, −8.1]	***	39.2 [17.9, 60.5]	***		

Δ Low–Med and Δ High–Med report differences in average predicted wealth (1,000 €) relative to the medium-complexity group, derived from GAMs using the delta method over the observed covariate distribution within each cluster × gender group. Models include age, country fixed effects, educational attainment, and the respective ideal-typical trajectory indicator (or all indicators jointly in the last row). Baseline Δ values (without trajectory controls) are reported in Table 6. β trajectory reports the parametric coefficient of the trajectory indicator in the GAM. Complexity levels: low (≤ − 0.5 SD), medium (− 0.5 to + 0.5 SD), high (≥ + 0.5 SD). 95% confidence intervals in brackets. Sig.:. *p* < 0.10, * *p* < 0.05, ** *p* < 0.01, *** *p* < 0.001

Data: SHARE W1-W7. *N* = 9,768

The general finding is that, across all contexts, the complexity–wealth gradient is only modestly affected by conditioning on qualitative trajectory patterns.

In the Scandinavian cluster, the high-complexity penalty among men attenuates from − 15k in the baseline model (Table 6) to Δ High–Med = − 12k once all ideal-typical trajectories and fertility are added, remaining statistically significant. The modern trajectory has a positive parametric association with wealth (β = +21k, *p* < 0.05) and accounts for a modest portion of the high-complexity penalty when added alone, but the high-end gradient persists in the full model. Among Scandinavian women, the low-complexity penalty (Δ Low–Med = − 38k) is remarkably stable across all specifications and is not attenuated by any individual trajectory pattern. The modern and dissolution trajectories both carry substantial independent associations with wealth (β = +29k and − 45k respectively, both *p* < 0.001), but these operate alongside rather than in place of the complexity gradient.

**Table 6:** Differences in average predicted wealth (1,000 €) relative to medium family complexity, baseline and adjusted for education

		Baseline				Adjusted for education			
		Δ Low–Med		Δ High–Med		Δ Low–Med		Δ High–Med	
Scandinavian	Men	−15.4 [−35.3, 4.5]		−16.0 [−30.8, −1.3]	*	−15.0 [−34.4, 4.4]		−15.4 [−29.8, −1.0]	*
	Women	−36.9 [−51.5, −22.3]	***	−5.2 [−17.8, 7.5]		−36.5 [−50.1, −23.0]	***	−5.4 [−17.1, 6.3]	
Continental	Men	−20.2 [−31.9, −8.4]	***	10.6 [−1.4, 22.5]		−21.3 [−31.4, −11.3]	***	10.2 [−0.4, 20.7]	
	Women	−35.8 [−46.9, −24.7]	***	−16.7 [−29.3, −4.2]	**	−37.3 [−47.8, −26.9]	***	−17.3 [−29.2, −5.4]	**
Southern	Men	−8.2 [−19.2, 2.8]		15.8 [0.2, 31.3]	*	−6.5 [−17.2, 4.1]		17.3 [2.3, 32.4]	*
	Women	−14.1 [−23.1, −5.0]	**	39.6 [19.8, 59.4]	***	−17.2 [−26.7, −7.8]	***	40.4 [19.9, 60.9]	***

Baseline models control for country and age at the interview. Adjusted models further control for educational attainment

Complexity levels: low complexity (≤ − 0.5 SD), medium complexity (between − 0.5 and + 0.5 SD), and high complexity (≥ + 0.5SD)

Countries in clusters: Scandinavia (Sweden, Denmark); Continental (Austria, Germany, France, Switzerland, Belgium); Southern (Spain, Italy, Greece)

Data: SHARE W1-W7. *N* = 30,509. Full tabular results in Table A3-A4-A5

In the Continental cluster, the low-complexity penalty for men (Δ Low–Med = − 24k) and both penalties for women (Δ Low–Med = − 40k, Δ High–Med = − 19k) persist — and if anything strengthen slightly relative to the baseline model (− 21k, − 37k, and − 17k respectively; Table 6) — once all trajectory controls are introduced. Dissolution is strongly and negatively associated with women’s wealth (β = −36k, *p* < 0.001), confirming it as an important and independent predictor, but it does not account for the complexity–wealth gradient at either end of the distribution.

In the Southern cluster, the complexity–wealth gradient is virtually unaffected by trajectory controls for both men and women. The high-complexity advantage for women (Δ High–Med = + 39k) and the corresponding advantage for men (Δ High–Med = + 16k) remain stable and statistically significant across all specifications, essentially unchanged from the baseline model (+ 40k and + 17k respectively; Table 6), reinforcing the interpretation that the positive gradient in this context reflects compositional selection rather than specific trajectory configurations.

### Robustness Checks

First, we tested the sensitivity of the GAM results by running the adjusted models with additional controls for employment history and receipt of inheritance (Figure A1). Results were in line with the main analyses. In addition, we re-estimated models using two alternative transformations of wealth: a rank-based specification (percentile rank of net wealth) and the inverse hyperbolic sine of wealth, which better accommodates zero and highly skewed values. Across both transformations, results closely resembled those of the main analyses. See Figures A2 and A3.

Second, to justify the aggregation of countries into three clusters, we performed a meta-analytic assessment of the country-specific OLS complexity–wealth associations. Random-effects models indicate substantial heterogeneity across countries, and a meta-regression with the three clusters as moderator shows that this heterogeneity is partly structured along our institutional grouping, with cluster membership explaining nearly half of the between-country variance. See Tables A7 and A8 and Figure A4 in the Appendix.

## Discussion and Conclusions

### Summary of Findings and Limitations

This study addressed a gap in the existing literature by examining how the complexity of entire family trajectories, rather than isolated demographic events, relates to personal net wealth in later life. Prior research has documented the wealth consequences of specific events such as marriage, divorce, and cohabitation, but has largely done so within single-country settings and without attending to how the cumulative, ordered, and often reversible nature of family transitions over the life course shapes long-term economic outcomes (Killewald et al., 2023; Lersch, 2017; Vespa & Painter, 2011; Kapelle et al., 2025). This study examined how the complexity of individual family trajectories — understood as the number, diversity, and volatility of family transitions experienced between ages 15 and 50 — relates to personal wealth in later life. Using retrospective and prospective data from the SHARE survey for cohorts born between the 1930 s and 1960 s across ten European countries, we analysed whether this association varies across three institutional clusters — Scandinavian, Continental, and Southern — and whether it differs by gender.

First, we showed that family complexity is unevenly distributed across institutional contexts and tightly linked to qualitative trajectory patterns. High complexity is common in Scandinavian countries and sizeable in Continental ones, but rare in Southern countries, where most individuals fall into the medium-complexity group. Across all clusters, high complexity is characterised by the virtual disappearance of traditional trajectories and the dominance of modern and dissolution patterns, while low complexity is strongly associated with traditional and, in Southern countries, postponement trajectories. Within each cluster, however, the same level of complexity maps onto different qualitative configurations, so that the meaning of a given complexity score varies across institutional contexts.

Second, generalised additive models show that the association between family complexity and wealth is neither linear nor uniform across contexts or sex. In Scandinavian and Continental countries, predicted wealth peaks at moderate levels of complexity and is lower at both the low and the high end of the distribution; the pattern is however gendered: in Scandinavian countries, the high-end penalty is more pronounced among men, while women face a sharper disadvantage at low complexity; in Continental countries, women face penalties at both extremes whereas men show a significant penalty only at low complexity, with no significant association at the upper end, once education is controlled. In Southern countries, the gradient is monotonically positive for both sexes, with high-complexity individuals — and especially women — holding substantially more wealth than those with low or medium complexity. Educational attainment attenuates these patterns but does not eliminate them, indicating that sorting into complex trajectories explains part, but not all, of the complexity–wealth nexus. Moreover, women’s wealth is more strongly and consistently linked to family complexity than men’s across virtually all institutional contexts, though the form of this association varies by cluster. In Continental countries, women face wealth penalties at both ends of the complexity distribution, consistent with cumulative disadvantage accounts in which women’s labour market trajectories, pension accumulation, and asset ownership are more closely tied to family arrangements than men’s (Ruel & Hauser, 2013; Nutz & Gritti, 2022).

Third, supplementary analyses including trajectory indicators as parametric controls show that the complexity–wealth gradient is largely irreducible to qualitative differences in family sequencing across all three clusters. In the Scandinavian cluster, the high-complexity penalty among men and the low-complexity penalty among women both persist after all trajectory controls are introduced, indicating that the gradient reflects within-trajectory differentiation over and above specific biographical configurations. In the Continental cluster, the low-complexity penalty for men and both penalties for women similarly survive conditioning on all trajectory patterns. Dissolution trajectories carry a large independent negative association with women’s wealth in both the Continental and Scandinavian clusters, but does not account for the complexity–wealth gradient at either end of the distribution.

Overall, three findings stand out. First, the complexity–wealth relationship is non-linear, with both ends of the complexity spectrum associated with lower wealth in Scandinavian and Continental countries, though via opposite mechanisms: cumulative disruption and instability at the high end, constrained joint accumulation and limited investment capacity at the low end. Second, the association is strongly gendered and context-specific: women’s wealth is more tightly coupled to family trajectories across all clusters, and the form of this dependence varies substantially by institutional setting. Third, the observed gradient cannot be reduced to the qualitative content of family biographies: controlling for whether individuals followed traditional, modern, dissolution, or postponement trajectories leaves the non-linear complexity–wealth profile essentially intact. Together, these findings suggest that demographic diversification leaves lasting imprints on material conditions in later life — imprints that event-centred analyses cannot fully capture.

The study is not without limitations. The cross-sectional measurement of wealth and the assumption of equal sharing within couples constrain what can be inferred about gender differences in wealth. Because we derive individual-level wealth for partnered respondents under an equal-sharing assumption, wealth gaps by gender are directly observable only among singles. Our estimates of the gendered complexity–wealth association are therefore likely conservative. Furthermore, although we explicitly condition on educational attainment as a key confounder, other dimensions of socioeconomic status — including social class of origin, occupational prestige, and early-life wealth transfers — may simultaneously shape both family behaviour and wealth accumulation. To the extent that these factors are correlated with both complexity and wealth, our estimates may still partly reflect selection processes that we cannot fully account for. Designs that exploit sibling variation would allow for a more precise disentanglement of family-of-origin effects from the independent contribution of family trajectories. Most importantly, the study’s design does not permit causal inference. Although family trajectories are measured prior to the observation of wealth, which helps reduce concerns about reverse causality, unobserved heterogeneity and feedback loops between family behaviour and economic resources remain plausible. Simultaneous analyses of both family processes and wealth accumulation across the life course would be needed to make progress on the causal question.

### Discussion and Conclusion

This study offers an integrated account of how demographic diversification intersects with wealth stratification in later life. While trajectory-based findings are broadly consistent with the event-centred literature — insofar as marriage and partnership stability emerge as wealth-enhancing and dissolution as wealth-depleting — they also reveal patterns that single-event approaches cannot capture and add relevant nuances to the complexity-wealth association.

The dominant theoretical expectation has been that high family complexity is economically costly: repeated transitions, dissolution and reconstitution of households, and sustained uncertainty are all expected to hinder long-term financial planning and deplete wealth (Avellar & Smock, 2005; Killewald et al., 2017; Lersch et al., 2022). Our findings contradict this partly, and reveal unanticipated patterns: in Southern countries complexity enhances wealth, in Scandinavian and Continental contexts low-complexity individuals hold less wealth than those with intermediate complexity. Very low complexity may reflect prolonged singlehood or early union formation without subsequent progression, configurations that forgo the economies of scale, joint investment opportunities, and risk-sharing associated with stable partnership (Bennett, 2013; Kapelle & Lersch, 2020) and that limit the capacity for sustained financial commitments such as homeownership. Both ends of the complexity spectrum thus generate wealth disadvantages, but through opposite hypothesized mechanisms: high complexity through disruption and instability, low complexity through constrained opportunity.

The marked cross-national heterogeneity in the complexity–wealth gradient calls for contextual interpretation. While complexity carries different meanings across different contexts, complexity exerts an influence on wealth also independently of the specific demographic patterns. Contrary to the expectation that individualising policy contexts might buffer the economic costs of complexity, our findings suggest that the widespread normalisation of complex biographies does not prevent their cumulative economic toll, particularly for men in Scandinavian countries. Where family diversification is widespread and no longer selects strongly for specific social groups, high complexity may still carry cumulative economic costs that generous welfare provisions only partially offset. The observation that complexity is most penalising precisely where it is most common and socially accepted suggests that the economic mechanisms embedded in family transitions — disrupted savings trajectories, housing instability, reduced joint investment horizons — operate regardless of normative accommodation (Hofäcker & Chaloupková, 2014). In Southern countries, where complexity remains limited and constrained by familistic norms, the positive gradient likely reflects a different selection process: those who do experience high complexity — especially women — may disproportionately have stronger labour market attachment and greater economic autonomy than their low-complexity counterparts. Disentangling institutional buffering from compositional selection would require richer individual-level data on employment histories, pension rights, and asset ownership, and remains an important avenue for future research.

Educational attainment attenuates the complexity–wealth association across all clusters but does not eliminate it. Educational sorting partly explains the gradient — more educated individuals tend to follow less turbulent family biographies, and education itself reflects early-life socioeconomic advantages including family-of-origin wealth (Killewald et al., 2017; Hansen & Toft, 2021; Zimmermann & Konietzka, 2018). Yet the persistence of the association net of education confirms that family trajectories add an independent layer of stratification on top of already unequal starting positions, one that unfolds gradually across the adult life course and is particularly consequential for women.

From a policy perspective, the non-linear association has implications at both ends of the complexity spectrum. The high-end penalty — especially for women in Continental countries, where it is closely tied to dissolution — underscores the importance of measures supporting economic recovery after dissolution. The low-end penalty, meanwhile, suggests that biographical immobility may also warrant policy attention, particularly in contexts where access to wealth-building opportunities such as homeownership is tightly coupled with partnered living. More broadly, our findings suggest that policies aimed at reducing the economic vulnerability associated with family complexity need to address not only the consequences of family disruption, but also the structural conditions that channel some individuals into trajectories with limited opportunities for wealth building.

## Supplementary Information

Below is the link to the electronic supplementary material.

Supplementary material 1 (DOCX 423.3 kb)

## Footnotes

1 Given the very low and selective prevalence of widowhood in the 15–50 observational window, we do not attempt to distinguish its wealth consequences from those of divorce in the pattern typology and instead capture such trajectories via higher levels of complexity.

2 We prefer this theory-based approach to a cluster analysis of typical trajectories, as used by Gruijters et al. (2023) and Kapelle and Vidal (2022). As discussed in recent contributions (Helske et al., 2024), the cluster approach can be problematic, especially when the resulting clusters are used as independent variables in regression models: the use of crisp clustering algorithms forces the relationship between trajectories and the dependent variable to be fully explained by assigning trajectories to mutually exclusive groups, ignoring within-cluster heterogeneity.
