# Supplementary material for: Family Complexity and Later-Life Wealth: A Comparative Perspective Across European Countries
Source: Eur J Popul. 2026 Aug 20;42(1):24. doi: 10.1007/s10680-026-09781-x (PMC13683266; doi:10.1007/s10680-026-09781-x)
Supplement: Supplementary file 1 [file 10680_2026_9781_MOESM1_ESM.docx]

FAMILY COMPLEXITY AND LATER-LIFE WEALTH:

A COMPARATIVE PERSPECTIVE ACROSS EUROPEAN COUNTRIES

ONLINE APPENDIX

**Table A1 –** Stepwise sample selection process. SHARE W1-W7.

|  | Individuals | Percentage of initial sample |
| --- | --- | --- |
| Panel W1-W7 | 139,012 |  |
| Country selection | 74,478 | 53% |
| Birth cohort 1932-1962 | 59,050 | 42% |
| Single or coupled individuals | 58,852 | 42% |
| Complete cases | 30,509 | 21% |

**Table A2 –** Descriptive statistics. SHARE W1-W7. N 30,509

|  | Scandinavian N 6,089 | Continental N 14,652 | Southern N 9,768 |
| --- | --- | --- | --- |
| Countries | Sweden, Denmark | Austria, Germany, France, Switzerland, Belgium | Spain, Italy, Greece |
| Average age | 61.90 | 61.60 | 62.60 |
| % Women | 51.30 | 48.30 | 47.30 |
| % Low education | 25.60 | 24.00 | 66.60 |
| % Medium education | 31.50 | 40.80 | 17.90 |
| % High education | 42.90 | 35.20 | 15.50 |
| Avg. complexity | 0.17 | 0.14 | 0.12 |
| % Traditional | 21.40 | 42.40 | 70.60 |
| % Modern | 56.30 | 27.80 | 8.40 |
| % Postponement | 5.90 | 7.90 | 10.10 |
| % Dissolution | 21.20 | 19.40 | 4.90 |
| Avg. # children | 2.25 | 2.01 | 2.01 |
| Average years of employment | 33.30 | 32.70 | 28.00 |
| Ever received inheritance/gift | 35.20 | 31.00 | 14.70 |
| Mean net wealth (€k) | 160.90 | 191.60 | 154.70 |

**Table A3 –** Generalized additive model of wealth accumulation at 50+. Data: SHARE W1-W7. Scandinavian cluster.

|  | Men | | | | Women | | | |
| --- | --- | --- | --- | --- | --- | --- | --- | --- |
|  | Baseline | | Adjusted | | Baseline | | Adjusted | |
|  | β (SE) | p | β (SE) | p | β (SE) | p | β (SE) | p |
| Complexity (smooth) | F = 2.12 edf = 6.15 | 0.039 | F = 2.73 edf = 5.79 | 0.009 | F = 7.87 edf = 4.50 | <0.001 | F = 6.70 edf = 3.51 | <0.001 |
| Intercept | 123.751 (34.505) | <0.001 | 73.906 (35.226) | 0.036 | 115.424 (31.351) | <0.001 | 32.778 (33.332) | 0.325 |
| Age | 0.515 (0.538) | 0.338 | 0.706 (0.533) | 0.185 | 0.416 (0.495) | 0.400 | 1.106 (0.500) | 0.027 |
| Denmark | 22.348 (7.932) | 0.005 | 16.872 (8.187) | 0.039 | 31.386 (7.165) | <0.001 | 26.039 (7.191) | <0.001 |
| Education: Medium |  |  | 23.118 (10.595) | 0.029 |  |  | 39.572 (9.925) | <0.001 |
| Education: High |  |  | 83.089 (10.263) | <0.001 |  |  | 67.823 (9.193) | <0.001 |
| N | 2968 |  | 2968 |  | 3121 |  | 3121 |  |
| Note: Baseline models control for age and country fixed effects. Adjusted models additionally control for educational attainment. For parametric terms, estimates are regression coefficients (β) with standard errors (SE). For the smooth term (Complexity), F is the approximate F-statistic and edf the estimated degrees of freedom of the penalised spline; edf ≈ 1 indicates an approximately linear association, higher values indicate increasing non-linearity. | | | | | | | | |

**Table A4 –** Generalized additive model of wealth accumulation at 50+. Data: SHARE W1-W7. Continental cluster.

|  | Men | | | | Women | | | |
| --- | --- | --- | --- | --- | --- | --- | --- | --- |
|  | Baseline | | Adjusted | | Baseline | | Adjusted | |
|  | β (SE) | p | β (SE) | p | β (SE) | p | β (SE) | p |
| Complexity (smooth) | F = 2.14 edf = 3.36 | 0.074 | F = 1.06 edf = 2.28 | 0.423 | F = 6.02 edf = 4.74 | <0.001 | F = 5.57 edf = 4.07 | <0.001 |
| Intercept | 123.843 (28.354) | <0.001 | 41.508 (29.443) | 0.159 | 119.775 (26.518) | <0.001 | 15.831 (27.539) | 0.565 |
| Age | 0.189 (0.435) | 0.664 | 0.414 (0.430) | 0.335 | 0.007 (0.411) | 0.987 | 0.790 (0.408) | 0.053 |
| Germany | 19.568 (10.140) | 0.054 | 10.967 (9.996) | 0.273 | 10.806 (9.290) | 0.245 | 4.731 (9.176) | 0.606 |
| France | 81.880 (10.815) | <0.001 | 100.652 (10.777) | <0.001 | 92.300 (9.563) | <0.001 | 102.882 (9.444) | <0.001 |
| Switzerland | 149.040 (11.738) | <0.001 | 145.554 (11.549) | <0.001 | 147.053 (11.112) | <0.001 | 148.096 (10.917) | <0.001 |
| Belgium | 96.395 (10.043) | <0.001 | 102.170 (10.097) | <0.001 | 88.837 (9.145) | <0.001 | 85.740 (9.029) | <0.001 |
| Education: Medium |  |  | 39.927 (9.025) | <0.001 |  |  | 40.199 (7.627) | <0.001 |
| Education: High |  |  | 129.323 (9.098) | <0.001 |  |  | 124.376 (8.000) | <0.001 |
| N | 7572 |  | 7572 |  | 7080 |  | 7080 |  |
| Note: Baseline models control for age and country fixed effects. Adjusted models additionally control educational attainment. For parametric terms, estimates are regression coefficients (β) with standard errors (SE). For the smooth term (Complexity), F is the approximate F-statistic and edf the estimated degrees of freedom of the penalised spline; edf ≈ 1 indicates an approximately linear association, higher values indicate increasing non-linearity. | | | | | | | | |

**Table A5 –** Generalized additive model of wealth accumulation at 50+. Data: SHARE W1-W7. Southern cluster.

|  | Men | | | | Women | | | |
| --- | --- | --- | --- | --- | --- | --- | --- | --- |
|  | Baseline | | Adjusted | | Baseline | | Adjusted | |
|  | β (SE) | p | β (SE) | p | β (SE) | p | β (SE) | p |
| Complexity (smooth) | F = 2.53 edf = 3.10 | 0.047 | F = 1.02 edf = 2.93 | 0.493 | F = 6.65 edf = 2.83 | <0.001 | F = 2.02 edf = 3.45 | 0.093 |
| Intercept | 242.430 (25.306) | <0.001 | 153.279 (25.930) | <0.001 | 218.323 (25.494) | <0.001 | 103.539 (26.352) | <0.001 |
| Age | -1.170 (0.389) | 0.003 | -0.114 (0.393) | 0.771 | -0.663 (0.395) | 0.094 | 0.807 (0.402) | 0.045 |
| Italy | -12.946 (6.769) | 0.056 | -15.045 (6.711) | 0.025 | -20.931 (7.265) | 0.004 | -25.558 (7.153) | <0.001 |
| Greece | -33.401 (7.495) | <0.001 | -51.721 (7.531) | <0.001 | -52.489 (7.729) | <0.001 | -64.728 (7.661) | <0.001 |
| Education: Medium |  |  | 63.643 (7.799) | <0.001 |  |  | 65.151 (8.458) | <0.001 |
| Education: High |  |  | 97.205 (8.159) | <0.001 |  |  | 123.409 (9.129) | <0.001 |
| N | 5150 |  | 5150 |  | 4618 |  | 4618 |  |
| Note: Baseline models control for age and country fixed effects. Adjusted models additionally control educational attainment. For parametric terms, estimates are regression coefficients (β) with standard errors (SE). For the smooth term (Complexity), F is the approximate F-statistic and edf the estimated degrees of freedom of the penalised spline; edf ≈ 1 indicates an approximately linear association, higher values indicate increasing non-linearity. | | | | | | | | |

**Figure A1** – Average marginal effects (AME) of standardized family complexity on wealth accumulation, across country clusters.

All models control for country, age and age squared at the interview. Adjusted models further control for educational attainment. Full adjusted models further control for years of employment and receipt of inheritances.
Countries in clusters: Scandinavia (Sweden, Denmark); Continental (Austria, Germany, France, Switzerland, Belgium); Southern (Spain, Italy, Greece).
Data: SHARE W1-W7. N = 30,509

**Figure A2** – Average marginal effects (AME) of standardized family complexity on wealth accumulation (rank), across country clusters.

All models control for country, age and age squared at the interview. Adjusted models further control for educational attainment. Countries in clusters: Scandinavia (Sweden, Denmark); Continental (Austria, Germany, France, Switzerland, Belgium); Southern (Spain, Italy, Greece).
Data: SHARE W1-W7. N = 30,509

**Figure A3** – Average marginal effects (AME) of standardized family complexity on wealth accumulation (IHS-transformed), across country clusters.

All models control for country, age and age squared at the interview. Adjusted models further control for educational attainment. Countries in clusters: Scandinavia (Sweden, Denmark); Continental (Austria, Germany, France, Switzerland, Belgium); Southern (Spain, Italy, Greece).
Data: SHARE W1-W7. N = 30,509

**Figure A4** – Forest plot of effect of standardized family complexity on wealth across countries.

All models control for country, age and age squared at the interview. Data: SHARE W1-W7. N = 30,509

**Table A7 –** Summary statistics from meta-analysis of effect of standardized family complexity on wealth across countries. Data: SHARE W1-W7. N = 30,509

|  | tau2 | I2 | Q | R2 | QM | df_QM | p_QM |
| --- | --- | --- | --- | --- | --- | --- | --- |
| Random-effects model | 34.58 | 66.6 | 25.82 |  |  |  |  |
| Random-effects model + country cluster | 18.81 | 50.1 | 13.52 | 45.6 | 6.28 | 2 | 0.043 |

**Table A8 –** Summary statistics from meta-analysis of effect of standardized family complexity on wealth across countries. Data: SHARE W1-W7. N = 30,509

| Group | k | beta | se | ci_lb | ci_ub | tau2 | I2 |
| --- | --- | --- | --- | --- | --- | --- | --- |
| All countries | 10 | -2.99 | 2.35 | -7.61 | 1.62 | 34.58 | 66.6 |
| Scandinavian | 2 | -7.53 | 4.36 | -16.08 | 1.02 | 26.54 | 69.3 |
| Continental | 5 | -4.90 | 2.10 | -9.02 | -0.78 | 2.92 | 12.8 |
| Southern | 3 | 4.83 | 5.09 | -5.15 | 14.80 | 53.67 | 69.9 |
